# Decursinol Angelate Inhibits LPS-Induced Macrophage Polarization through Modulation of the NFκB and MAPK Signaling Pathways

**DOI:** 10.3390/molecules23081880

**Published:** 2018-07-27

**Authors:** Salman Ul Islam, Jung Ho Lee, Adeeb Shehzad, Eun-Mi Ahn, You Mie Lee, Young Sup Lee

**Affiliations:** 1 School of Life Sciences, BK21 Plus KNU Creative BioResearch Group, Kyungpook National University, Daegu 41566, Korea; salman2013@knu.ac.kr (S.U.I.); quiet93@dgist.ac.kr (J.H.L.); 2 Department of Biomedical Engineering and Sciences, School of Mechanical and Manufacturing Engineering, National University of Sciences and Technology, Islamabad 44000, Pakistan; adeeb.shehzad@smme.nust.edu.pk; 3 Division of Biotechnology and Convergence, Daegu Haany University, Daegu 38610, Korea; ahnEM@dhu.ac.kr; 4 College of Pharmacy, National Basic Research Laboratory of Vascular Homeostasis Regulation, Kyungpook National University, Daegu 41566, Korea; lym@knu.ac.kr

**Keywords:** inflammation, cytokines, MAP kinase, NFκB, decursinol angelate

## Abstract

Inflammation is considered the root cause of various inflammatory diseases, including cancers. Decursinol angelate (DA), a pyranocoumarin compound obtained from the roots of *Angelica gigas*, has been reported to exhibit potent anti-inflammatory effects. In this study, the anti-inflammatory effects of DA on the MAP kinase and NFκB signaling pathways and the expression of pro-inflammatory cytokines were investigated in phorbol 12-myristate 13-acetate (PMA)-activated human promyelocytic leukemia (HL-60) and lipopolysaccharide (LPS)-stimulated macrophage (Raw 264.7) cell lines. PMA induced the activation of the MAP kinase-NFκB pathway and the production of pro-inflammatory cytokines in differentiated monocytes. Treatment with DA inhibited the activation of MAP kinases and the translocation of NFκB, and decreased the expression and exogenous secretion of IL-1β and IL-6. Furthermore, LPS-stimulated Raw 264.7 cells were found to have increased expression of M1 macrophage-associated markers, such as NADPH oxidase (NOX) and inducible nitric oxide synthase (iNOS), and the M2 macrophage-associated marker CD11b. LPS also activated pro-inflammatory cytokines and Erk-NFκB. Treatment with DA suppressed LPS-induced macrophage polarization and the inflammatory response by blocking Raf-ERK and the translocation of NFκB in Raw 264.7 cells. Treatment with DA also inhibited the expression of pro-inflammatory cytokines, such as IL-1β and IL-6, NOX, and iNOS in Raw 264.7 cells. These results suggest that DA has the potential to inhibit macrophage polarization and inflammation by blocking the activation of pro-inflammatory signals. These anti-inflammatory effects of DA may contribute to its potential use as a therapeutic strategy against various inflammation-induced cancers.

## 1. Introduction

Inflammation, the immediate response of the body to a damage, is triggered by noxious stimuli and conditions, such as infection and tissue injury [1]. Inflammation is a complex physiological process that can promote cancer development through abnormal gene regulation and signal transduction. There is growing evidence that many cancers are initiated by infections, and it has been estimated that among 1.2 million cases of cancer annually, 15% can be due to infections [2,3,4,5]. Within the host, persistent infections induce chronic inflammation, which subsequently, through multiple steps like generation of reactive oxygen and nitrogen species, DNA damage, and permanent genomic alterations (point mutations, deletions, or rearrangements), leads to cancer [6,7]. It is widely accepted that there is a causal relationship between inflammation, innate immunity and cancer; however, many underlying molecular and cellular mechanisms are still not clear. Tumor-associated macrophages (TAMs), which are found in the tumor microenvironment, are extremely differentiated cells derived from circulating monocytes. They exhibit pro-cancerous effects that are often related to tumor growth and invasion in cancer patients with poor clinical outcomes [8]. TAM activation or macrophage polarization by inflammatory cytokines (IFN-γ, TNF, and IL-12), anti-inflammatory cytokines (IL-4 and IL-10), or bacterial lipopolysaccharide (LPS) is the primary process involved in inflammation-associated cancers. This process results in the loss of homeostatic tissue architecture followed by immune suppression [9,10]. There is compelling evidence that immune responses are mediated by two main phenotypes of macrophages; M1 (classical) and M2 (alternative) [11,12]. M1 macrophages are activated by LPS, IFN-γ, and TNF-α, and are associated with the expression of cytokines such as IL-1β, IL-6, IL-9, IL-13, and inducible nitric oxide synthase (iNOS) in cancer patients [13,14]. The alternative M2 macrophages are associated with tumor progression and cell survival in cancer patients. Conversely, M2 macrophages have been reported to inhibit inflammation by producing anti-inflammatory cytokines, such as IL-4 and IL-10, that reduce the expression of iNOS and T cell proliferation [13,14]. M2 macrophages also mediate the disruption of the extracellular matrix (ECM) through increased vascularization [15].

Leukemia is a cancer of blood-forming cells usually present in the bone marrow. According to the American Cancer Society, a total of 62,130 new cases and 24,500 deaths caused by leukemia were expected in 2017 [16]. The role of inflammation has been demonstrated in leukemia. Toll-like receptor 4 (TLR4) is the main recognition receptor expressed in human monocytes and is abnormally regulated during inflammation [17]. Additionally, TLR4 has been found to be over-expressed in ulcerative colitis patients [18]. Upon activation, TLR4 augments macrophage activity through inflammatory cytokines, including TNF-α, IL-6, and IL-1β, the nuclear translocation of nuclear factor κB (NFκB), and the activation of mitogen-activated protein kinases (MAPKs) [19]. Studies have also reported that MAPK activation is involved in LPS-induced nitric oxide synthase (iNOS) expression, at least in part through the modulation of p38 and JNK in RAW 264.7 macrophages [20]. Activation of iNOS has also been shown to enhance inflammatory bowel disease by releasing excessive nitric oxide (NO), which erodes colon integrity via the synthesis of peroxynitrite, a potent oxidizing agent formed during the reaction of NO with the superoxide anion [21]. Inflammatory diseases are complicated, and detailed studies are needed to discover new treatment modalities.

Decursinol angelate (DA) is a pyranocoumarin compound isolated from the roots of the Korean medicinal herb *Angelica gigas Nakai*, which belongs to the family *Umbelliferae.* Traditionally, this herb has been used as an immune-booster in the treatment of gynecological disorders and anemia [22,23]. Several studies have characterized the anti-inflammatory, anticancer, and anti-angiogenic properties of decursin in various cancers, including myelogenous leukemia cell lines [24]. Shehzad et al. reported that decursin and DA are the potent candidates for the treatment of various chronic inflammatory diseases such as diabetic retinopathy, osteoclastogenesis, rheumatoid arthritis, cancers (leukemia, myeloma, breast, prostate, and cervical), allergy, hepatic fibrosis, and Alzheimer’s disease [25]. Studies have also reported that decursin inhibits matrix metallopeptidase 9 (MMP-9)-induced cytoskeletal rearrangement by suppressing phosphoinositide 3-kinase (PI3K), extracellular signal-regulated kinase (ERK), and NFκB activation in fibrosarcoma and breast cancer cell lines [26]. Decursin has also been shown to reduce the phosphorylation of ERK and JNK, but not p38 MAPK, in vascular endothelial growth factor (VEGF)-stimulated HUVECs [27].

Despite the well-documented anti-inflammatory effects of DA, the mechanisms underlying these effects are not yet fully understood. Therefore, the current study was conducted to elucidate the mechanism underlying the anti-inflammatory effects of DA on macrophage polarization. DA inhibited the differentiation and polarization of macrophages by suppressing the expression of inflammatory cytokines and the MAPK and NFκB pathways, as well as by reducing NADPH oxidase (NOX) and iNOS levels in LPS and TPA-induced inflammatory models.

## 2. Results

### 2.1. DA Inhibits Cytokine Production in PMA-Induced Macrophages

**Figure 1:**
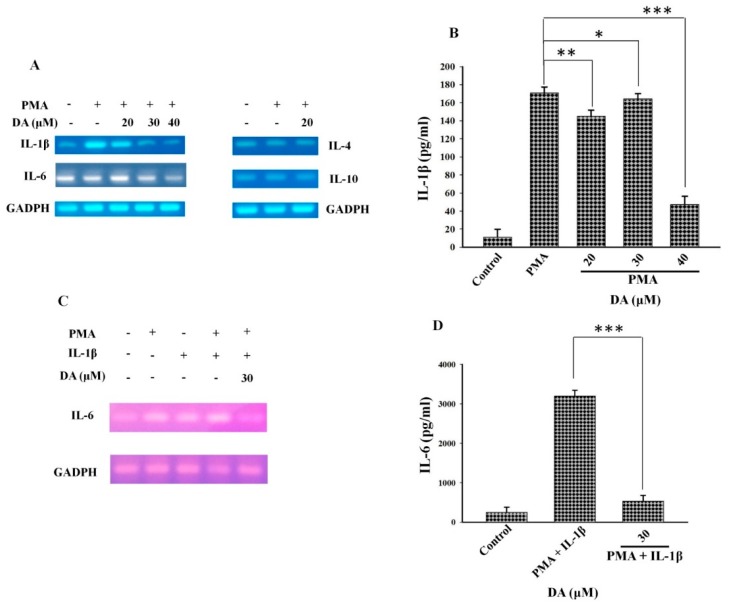
DA inhibits PMA-induced production of proinflammatory cytokines. (**A**) total RNA was extracted from the treated HL-60 cells using TRIZOL reagent and was reverse-transcribed into cDNA. The amplified products were analyzed through electrophoresis on a 1% agarose gel and visualized using an EcoDye™ Nucleic Acid Staining Solution. Images were then captured using Wise Capture I-1000 software (Daihan Scientific, Seoul, South Korea). DA inhibited pro-inflammatory cytokines in PMA-induced macrophages; (**B**) IL-1β exogenous secretion was measured through ELISA. A 96-well plate was coated with 10 μg/mL purified goat anti-IL-1β antibody. Cell culture supernatants from the control and treated groups were incubated in these plates for 1 h, and then a polyclonal rabbit anti-IL-1β antibody was added to the wells. HRP-conjugated goat anti-rabbit IgG was then added, and the cells were incubated for 30 min. ABTS solution was then added to the plate, and the signal that was developed was detected using a reference wavelength of 490 nm. Data are given as the mean ±  SD. * *p* < 0.05, ** *p* < 0.01, *** *p* < 0.001; (**C**) cells were treated with PMA overnight and then incubated with IL-1β, PMA, or 30 μM DA for the specified amount of time. RT-PCR was performed to analyze the expression of IL-6 at the mRNA level. PMA and IL-1β upregulated the mRNA expression of IL-6, which was effectively reduced by DA. GAPDH was used as the reference gene; (**D**) the exogenous secretion of IL-6 was analyzed through ELISA as described for IL-1β. PMA and IL-1β were found to exert a synergistic effect, while treatment with DA inhibited the exogenous secretion of IL-6. Data are given as the mean  ±  SD. *** *p* < 0.001.

Macrophages produce many pro-inflammatory cytokines; among those produced by M1 macrophages are IL-1, IL-4, IL-6, IL-12, and TNFα, while those produced by M2 macrophages include IL-10 and very low level of IL-12 [28]. Macrophages play a differential role in tumor growth and malignancy [28,29]. To evaluate the effects of DA treatment on cytokine production in PMA-stimulated macrophages, we analyzed the mRNA levels of IL-1β, IL-4, IL-6, and IL-10 through RT-PCR using RNA from DA-treated HL-60 cell lines as the template. PMA upregulated the mRNA levels of IL-1β and IL-6, whereas treatment with DA reduced the levels of IL-1β and IL-6 mRNA (Figure 1A). However, neither PMA nor DA affected the expression of IL-4 and IL-10 at the mRNA level (Figure 1A). We further evaluated the exogenous secretion of IL-1β after exposure to PMA and DA. As shown in Figure 1B, treatment with DA effectively inhibited the PMA-induced activation of IL-1β. Furthermore, to determine whether there is a synergistic effect between IL-1β and PMA that induces maximal IL-6 secretion, we exposed HL-60 cells to 5 ng/mL of IL-1. As shown in Figure 1C, the mRNA expression of IL-6 increased upon exposure to IL-1β and PMA alone, as well as their concomitant treatment, and decreased upon treatment with DA. Previous studies have shown that external stimuli, such as elevated temperature, induce the expression of IL-6. As shown in Figure 1D, IL-1β and PMA increased the expression and secretion of IL-6 into the culture media as measured through ELISA. DA treatment significantly reduced the IL-1β-induced secretion of IL-6 by PMA-induced differentiated cells. These data suggest that DA has the potential to inhibit the expression of pro-inflammatory cytokines in PMA-induced macrophages.

### 2.2. DA Inhibits PMA-Induced NFκB and MAPK Activation in Macrophages

**Figure 2:**
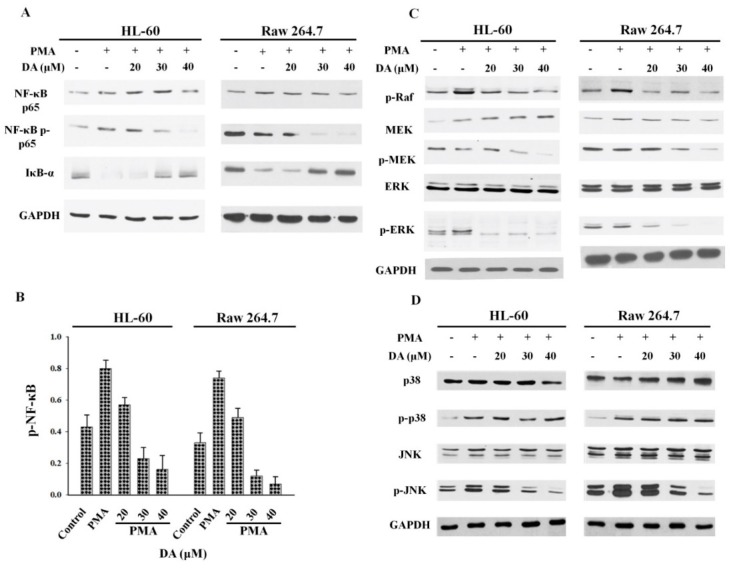
DA inhibits the NFκB and MAPK signaling pathways in PMA-induced macrophages. (**A**) treatment with DA abolished the activation of the NFκB pathway in PMA-induced macrophages. Cells were treated with PMA for 12 h in the dark and then incubated with different concentrations of DA (0, 20, 30, 40 μM) for the indicated amounts of time. Cell lysates were separated through 10% SDS-PAGE, and proteins were detected using an ECL Western blotting detection reagent. GAPDH was used as internal control; (**B**) NFκB phosphorylation was quantified using the software Image J. The results from three independent experiments are presented in a bar chart; (**C**) DA inhibits the activation of the ERK and JNK pathways. Cells were incubated with PMA or DA for the specified amounts of time, and the levels of total and phosphorylated proteins involved in the MAPK pathway were analyzed through Western blot. GAPDH was used as internal control; (**D**) cells were incubated with either PMA or DA for specified interval of time and MAPK pathway total and/or phosphorylated proteins were analyzed by Western blot.

The inflammatory cytokine IL-1 is responsible for the induction of IL-6 expression and the activation of MAPKs and NFκB in various inflammatory diseases [30]. NFκB is a transcription factor with pro-tumorigenic activity and is capable of evoking an inflammatory response. To study the anti-inflammatory mechanism of DA in macrophage polarization, HL-60 and Raw 264.7 cells were incubated with 100 ng/mL of PMA for 12 h and then treated with varying concentrations of DA (0, 20, 30, 40 μM) for 12 h. The levels of total and phosphorylated NFκB p65 subunit and IκBα were measured through Western blot. PMA treatment activated NFκB by inhibiting IκBα. However, DA treatment significantly suppressed the activation of the NFκB p65 subunit in both HL-60 and Raw 264.7 cells (Figure 2A). Quantitative analysis of the Western blot data also revealed that PMA increased the expression of NFκB p65 in both HL-60 and Raw 264.7 cell lines (Figure 2B). DA suppressed the PMA-induced activation of NFκB by stabilizing IκBα in macrophages. Proteins in the MAPK family are also involved in inflammatory diseases through their activation of inflammatory mediators. Drugs and other agents that inhibit this pathway are likely to have anti-inflammatory effects and may be of therapeutic interest. To determine the role of the MAPK pathway in PMA-induced macrophage polarization, we measured the expression levels of phosphorylated Raf, MEK, and ERK in cells that have been exposed to PMA and DA. PMA treatment increased the phosphorylation of Raf, MEK, and ERK in both HL-60 and Raw 264.7 cell lines (Figure 2C). However, DA treatment reduced phosphorylation and reversed the PMA-mediated activation of the MAPK pathway (Figure 2C). DA treatment did not significantly affect the p38/JNK pathway in HL-60 and Raw 264.7 cell lines (Figure 2D). These results indicate that DA may block the PMA-induced differentiation of macrophages by controlling the expression level of MAPK and NFκB.

### 2.3. Effect of DA on LPS-Induced M1 Macrophages

**Figure 3:**
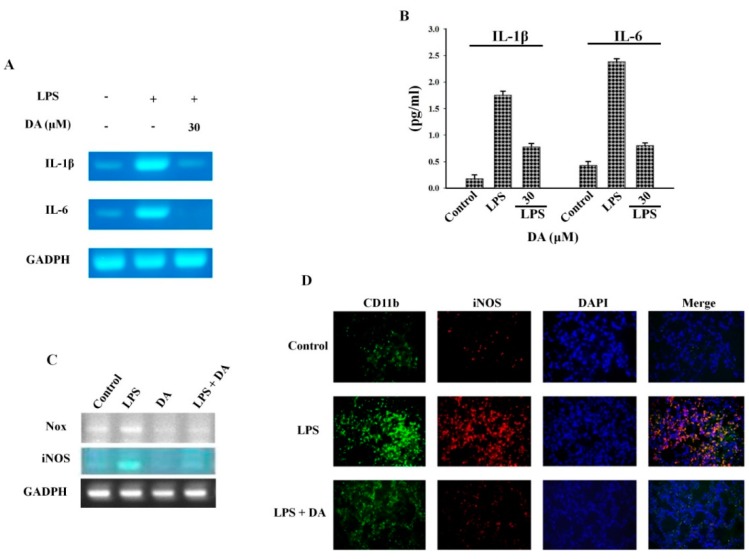
DA inhibits macrophage polarization. (**A**) mRNA levels of IL-1β and IL-6 were analyzed through RT-PCR after incubating the cells with LPS and 30 µM DA; (**B**) the exogenous secretion of IL-1β and IL-6 by LPS-stimulated cells was analyzed through ELISA. LPS stimulated the secretion of IL-1β and IL-6, which was significantly blocked by DA; (**C**) mRNA levels of NOX and iNOS in cells incubated with LPS with or without DA were analyzed through RT-PCR. (**D**) RAW 264.7 cells were seeded onto a 4-well chamber slide and cultured until 70% confluency was reached. The cells were then incubated with LPS with or without DA for the indicated amounts of time. Cells were fixed with 3.7% formaldehyde and then permeabilized by incubating them with 0.1% Triton X-100 for 4 min. Afterwards, the cells were incubated with anti-CD11b or anti-iNOS antibodies overnight and then incubated with FITC- or Alexa Flour 594-conjugated antibodies. Finally, the cells were incubated with DAPI mounting medium for 5–10 min and analyzed through confocal microscopy.

LPS has been reported to induce M1 macrophages, which results in higher levels of pro-inflammatory cytokines that dictate inflammatory T cell responses. Moreover, stimulation by LPS can transform M2 macrophages into M1 macrophages, thereby promoting inflammation [31]. To further elucidate the mechanisms underlying the activity of DA against LPS-induced macrophage polarization, Raw 264.7 cells were treated with bacterial LPS and the expression levels of inflammatory cytokines, such as IL-1β and IL-6, were measured. Treatment with LPS increased the expression of both IL-1β and IL-6 at the mRNA level, which was effectively reversed by treatment with 30 µM DA (Figure 3A). DA treatment also reduced the secretion of IL-1β and IL-6 by LPS-stimulated Raw 264.7 cells (Figure 3B). Since NOX-mediated iNOS production plays an important role in differentiation and macrophage polarization, we examined the mRNA expression of the M1 macrophage markers NOX and iNOS in LPS-stimulated Raw 264.7 cells through RT-PCR. As shown in Figure 3C, NOX and iNOS expression increased noticeably in cells treated with LPS (100 ng/mL). However, DA treatment abolished the LPS-induced NOX and iNOS expression (Figure 3C). In line with these results, we also performed immunofluorescence experiments to investigate the effect of DA on macrophage polarization. In these experiments, we used iNOS and CD11b as markers for the M1 and M2 phenotypes, respectively (Figure 3D). Microscopic analysis revealed that exposure to LPS increased the number of CD11b- and iNOS-positive cells as compared to the control set-up. DA treatment reduced the number of CD11b- and iNOS-positive cells and, consequently, inhibited macrophage polarization (Figure 3D). These results are consistent with those of the RT-PCR analysis, where DA was found to inhibit iNOS mRNA expression.

### 2.4. DA Suppresses LPS-Induced MAPK and NFκB Activation in Raw 264.7 Cells

**Figure 4:**
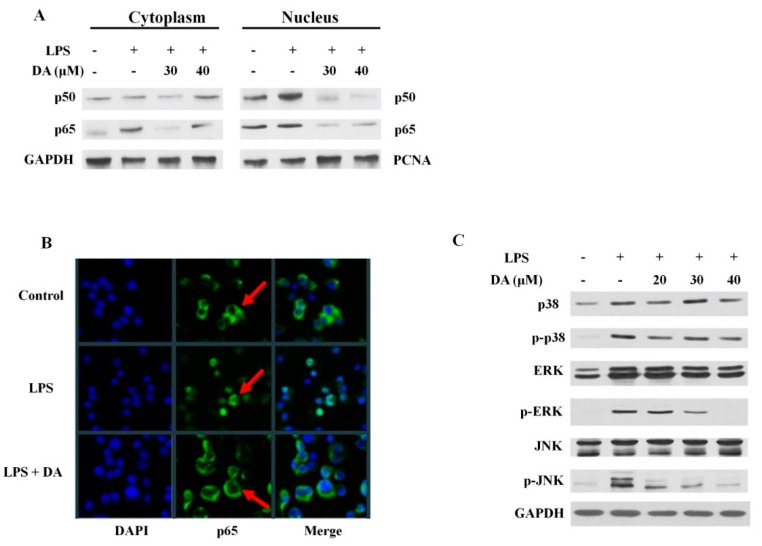
DA inhibits the activation of the NFκB and MAPK pathways by LPS in Raw 264.7 cells. (**A**) cells were stimulated with LPS and then incubated with or without DA for the indicated amounts of time. Cytoplasmic and nuclear fractions and total cell lysates were analyzed through a Western blot assay. DA inhibited the activation of the NFκB and MAPK pathways by LPS in Raw 264.7 cells; (**B**) nuclear localization of the p65 subunit of NFκB was analyzed through immunofluorescence. Briefly, cells were fixed with 3.7% formaldehyde and permeabilized by incubating them with 0.1% Triton X-100. The cells were incubated with anti-NFκB p65 antibodies overnight at 4 °C, and then with goat anti-rabbit IgG-FITC for 1 h at room temperature in the dark. Next, the cells were incubated with DAPI mounting medium for 10 min and analyzed using a confocal microscope. Fluorescent images of the cytoplasmic and nuclear fractions were merged to locate p65. DA inhibited the translocation of p65 into the nucleus; (**C**) DA inhibits the activation of the MAPK pathway. Cells were incubated with LPS with or without DA for the specified amounts of time, and the levels of total and/or phosphorylated proteins involved in the MAPK pathway were analyzed through Western blot.

There is compelling evidence that the activation of the MAPK and NFκb pathways is crucial for the induction of the expression of pro-inflammatory cytokines upon exposure to LPS. To determine whether LPS-induced macrophage polarization is mediated by the activation of NFκB (p50 and p65), Raw 264.7 cells were exposed to LPS and then treated with DA. To determine whether NFκB was translocated from the cytoplasm to the nucleus, we prepared cytosolic and nuclear fractions of Raw 264.7 cells. As shown in Figure 4A, LPS treatment induced the activation of NFκB, particularly of the subunits p50 and p65, which was effectively blocked by treatment with DA. Fluorescence microscopy is often used to identify drug targets by examining the sub-cellular localization of target proteins in inflammatory disorders. Therefore, we performed fluorescence microscopy to evaluate the translocation of NFκB from the cytoplasm to the nucleus. As shown in Figure 4B, LPS treatment resulted in the translocation of NFκB from the cytoplasm to the nucleus as indicated by the detection of the FITC-conjugated anti-NFκB antibody and the blue fluorescence of nuclear counterstain DAPI. In contrast, the translocation of NFκB was reduced in DA-treated cells (Figure 4B). The activation of MAPKs has been shown to play a central role in LPS-induced cytokine production during inflammation. Through the results we obtained, we have demonstrated the inhibitory effect of DA on the LPS-induced activation of the MAPK pathway (Figure 4C).

## 3. Discussion

It has long been established that inflammation and infections are strongly correlated with the development of various cancers and malignancies [2,3,4,5,8,10]. TAMs, which are recruited by inflammatory cytokines and chemokines at the tumor site, are the main players that cause tumor growth and inflammation through the promotion of angiogenesis, cancer cell invasion, and lymph node metastasis in the tumor microenvironment [32]. TAMs are derived from peripheral blood monocytes, and infiltration by TAMs has been linked to growth in various human tumors. They are thought to exert these effects by producing growth factors (VEGF) and proinflammatory molecules, such as IL-1β, IL-6, TNF-α, MMPs, and COX-2 [33]. Various agents, such as PMA and toxins like LPS, have previously been used to induce the differentiation, maturation, and activation of HL-60 and Raw 264.7 cells. Studies have shown that both PMA and LPS bind to the macrophage-specific cell surface receptors CD14 and CD11b, which ultimately results in the phosphorylation and activation of the MAPK/NFκB pathway and the production of inflammatory cytokines [31,34]. Various anti-inflammatory drugs, including the NSAIDs, have been found promising for treating the cancer. These drugs can alter the tumors themselves or the tumor microenvironment, potentially increasing cell death, decreasing migration, and increasing sensitivity of tumors to other therapies [35,36,37]. For instance, tolfenamic acid has been shown to exert potent anti-tumorigenic activities by regulating multiple molecular targets both in vitro and in nude mouse model [38,39,40,41,42]. DA has been reported to possess anti-inflammatory and anti-tumor activities, such as causing cell cycle arrest, suppressing angiogenesis, and inducing apoptotic cell death in various human tumors [22,23,24,26]. DA exerts its anti-inflammatory effects by modulating the activation of MMP-9, PI3K, ERK, NFκB, and VEGF in cancer cells [26]. Previously, it was reported that decursin inhibited MMP-9, nitric oxide production and cytokine (IL-8, MCP-1, IL-1β, and TNF-α), which was stimulated by pre-treatment with LPS, TLR-ligands, IL-1β, and TNF-α in RAW264.7 cells and THP-1 cells. The study suggested that decursin action was mediated through the inhibition of LPS-induced IκB phosphorylation and NF-κB translocation [43]. Therefore, we investigated the mechanism underlying the activity of DA against macrophage polarization in HL-60 and Raw 264.7 cells. DA significantly blocked macrophage polarization by inhibiting the production of proinflammatory cytokines and activating the MAPK and NFκB pathways. DA disrupted the interplay between cytokines and the MAPK/NFκB pathways in PMA- and LPS-activated macrophages.

Macrophages bidirectionally mediate events between inflammation and cancer development by displaying M1 and M2 phenotypic characteristics. Several reports have highlighted the differential role of M1 and M2 macrophages in various diseases. Macrophages of the M2 phenotype have been reported to promote tumor progression, whereas M1 macrophages have been shown to trigger insulin resistance and atherosclerosis [44]. The excessive production of proinflammatory cytokines such as IL1 and IL6, growth factors, chemokines, and proteases by macrophages results in precancerous lesions, tumor growth promotion, and metastasis. It has been reported that TAMs exhibit characteristics of M2 macrophages in the early stages of tumorigenesis and exert immunosuppressive effects [45,46]. Human HL-60 cells are widely used as an excellent model of the differentiation of monocytes to macrophages. When we treated HL-60 cells with PMA, they attached to the plate and differentiated into macrophages, which was accompanied by an increase in the expression of IL-1 and IL-6 (Figure 1B,C). Treatment with PMA also activated MAPK and NFκB in HL-60 and RAW 264.7 cells (Figure 2A,C). The activation of MAPKs and NFκB induces the transcription of pro-inflammatory cytokines, including IL1β, IL-6, and IL-12 [47,48]. The induction of IL-6 by IL1β is mediated by MAPKs and NFκB during inflammation. Our results are consistent with previous reports in showing that IL-1β increased the secretion of IL-6 in response to PMA (Figure 1C). DA effectively blocked the PMA-induced activation of the NFκB and MAPK pathways as well as the production of pro-inflammatory cytokines, such as IL-1β and IL-6, in HL-60 and RAW 264.7 cells.

The bacterial endotoxin LPS is a known agonist of TLR2 that activates the expression of proinflammatory cytokines and the phosphorylation of MAPKs and NFκB [49]. In addition, the activation of the MAPK and NFκB signaling cascades drive inflammation and macrophage polarization. The results of the present study show that the activation of MAPK and NFκB is involved in the modulation of NOX and iNOS expression in LPS-stimulated RAW 264.7 macrophages (Figure 3C). DA treatment not only blocked the differentiation of macrophages by inhibiting NOX-mediated iNOS generation, but also inhibited the activation of IL-1β and IL-6 in LPS-stimulated RAW 264.7 cells (Figure 3B). During macrophage differentiation, when there is an increase in the abundance of the cell surface marker CD11b upon translocation to the plasma membrane, NOX primarily regulates the production of ROS [50,51]. In LPS-induced signal transduction, ROS may serve as a secondary messenger, facilitating the regulation of downstream pathways such as NFκB and MAPK, which eventually promote the expression of proinflammatory genes [52]. DA treatment reduced the expression of the M1-specific marker iNOS and the M2-specific marker CD11b, thereby blocking the polarization of macrophages (Figure 3C). Given the differential role of MAPKs in macrophage polarization, we also highlight the fact that LPS-induced differentiation was mainly dependent on the ERK pathway, with negligible input from the p38 or JNK pathway (Figure 2D). On the other hand, NFκB translocation was blocked by DA treatment, suggesting that the potential anti-inflammatory effects of DA are mediated by its ability to regulate macrophage phenotype characteristics (Figure 4B).

Inflammation has been shown to be closely associated with an increase in the incidence of various diseases, including cancer. Interleukins (IL-1β/IL-6), which are key players in inflammation, mediate macrophage polarization by promoting the activity of the NFκB and MAPK signaling pathways and the overproduction of NOX and iNOS. Our results show that DA, which is isolated from the roots of *A. gigas*, has the potential to inhibit inflammatory responses during macrophage differentiation. This anti-inflammatory effect on macrophages is expected to contribute to the anti-cancer activity of DA. We found that treatment with DA suppressed macrophage polarization by inhibiting the production of inflammatory cytokines and free radicals and by activating the NFκB and MAPK pathways. These results suggest that DA might be of therapeutic importance in other inflammatory diseases, but its detailed mechanism of action needs to be investigated.

## 4. Materials and Methods

### 4.1. Chemicals and Reagents

DA with molecular weight 328 was isolated from the roots of *Angelicae gigas* at Daegu Hanny University, Daegu, Korea as described previously [22]. LPS (cat# L 2630) and PMA (cat# P1585) were purchased from Sigma-Aldrich (St. Louis, MO, USA). Mounting medium with DAPI (cat# H-1200) was obtained from Vector Laboratories, Inc. (Burlingame, CA, USA). Electrophoresis reagents and the Bio-Rad protein assay kit were purchased from Bio-Rad (Hercules, CA, USA). Antibodies were obtained from Santa Cruz Biotechnology (Dallas, TX, USA), Cell Signaling Technology (Danvers, MA, USA), Abcam (Cambridge, UK), and Life Technologies Corporation (Carlsbad, CA, USA). ECL Western blotting detection reagents and nitrocellulose membrane were obtained from Amersham Pharmacia Biotech (Little Chalfont, Buckinghanshire, UK). Superscript III First-Strand Synthesis System kit was purchased from Invitrogen Life Technologies (Waltham, MA, USA). Antibodies were obtained from Santa Cruz Biotech (p65 (sc-372), p50 (sc-1190), IκBα (sc-1643), p-Raf (sc-271929), ERK (sc-292838), p-ERK (sc-7383), JNK (sc-7345), p-JNK (sc-6254), p-38 (sc-271120), and p-p38 (sc-101759)) and Cell Signaling Technology (MEK (cat# 9122), p-MEK (cat# 9121), GAPDH (cat# 2118), and PCNA (cat# 13110)). All chemicals were stored and used according to the manufacturer’s instructions.

### 4.2. Cell. Culture and Treatment

Human leukemia HL-60 (ATCC #CCL-240) and mouse macrophage RAW 264.7 cells (ATCC #TIB-71) were cultured in Iscove’s Modified Dulbecco’s Medium (IMDM, Gibco #12440-053) and Dulbecco`s Modified Eagle Medium (DMEM, Gibco #11995-065), respectively. Both media were supplemented with 10% Fetal Bovine Serum (Gibco #16000-044) and 1% penicillin-streptomycin (Gibco #15140-122). Cell cultures were maintained in a humidified incubator containing 5% CO_2_ at 37 °C. HL-60 cells were treated with 100 nM PMA for 48 h to induce cellular differentiation into macrophages (M_0_). After differentiation, cells were treated with varying concentrations of DA (0, 20, 30, and 40 μM). The cells were then collected and washed with PBS to remove cell debris and particles. Similarly, Raw 264.7 macrophages were incubated for 24 h with LPS (1 μg/mL) with or without exposure to DA. 

### 4.3. Nuclear and Cytoplasmic Fractionation

Cells were cultured at a density of 2 × 10^5^ cells/mL, and once 70% confluency was reached, cells were pretreated with LPS and incubated with DA for 24 h. Cells were harvested using trypsin-EDTA, collected through centrifugation, and washed thrice with cold PBS. Fractions were prepared using a Nuclear Extraction Kit (Cayman Chemicals, Ann Arbor, MI, USA) according to the manufacturer’s protocol. Nuclear and cytosolic fractions were stored at −75 °C.

### 4.4. Reverse-Transcription Polymerase Chain Reaction (RT-PCR)

Total RNA was isolated from the cells using TRIZOL reagent (Invitrogen, Carlsbad, CA, USA) according to the manufacturer’s protocol. The Superscript III First-Strand Synthesis System kit was used to isolate and purify mRNA from total RNA according to the manufacturer’s instructions. Reverse transcription and subsequent cDNA amplification were performed with an Access RT-PCR Introductory System (Promega, Madison, WI, USA) in an Eppendorf Mastercycler. PCR cycle parameters were as follows: initial denaturation at 98 °C for 3 min, followed by 30–40 cycles of denaturation at 95 °C for 30 s, annealing at 55 °C for 30 s, and extension at 72 °C for 30 s, and a final extension step at 72 °C for 5 min. The sequences of the primers used were as follows: IL-1β forward, 5′-ACAGATGAAGTGCTCCTTCCA-3′, and IL-1β reverse, 5′-GTCGGAGATTCGTAGCTGGAT-3′; IL-4 forward, 5′-CGAGTTGACCGTAACAGACAT-3′, and IL-4 reverse, 5′-CGTCTTTAGCCTTTCCAAGAAG-3′; IL-6 forward, 5′-GGTACATCCTCGACGGCATCT-3′, and IL-6 reverse, 5′-GTGCCTCTTTGCTGCTTTCAC-3′; IL-10 forward, 5′-GCCTAACATGCTTCGAGATC-3′, and IL-10 reverse, 5′-CTCATGGCTTTGTAGATGCC-3′; and GADPH forward, 5′-AGGGCTGCTTTTAACTCTGGT-3′, and GADPH reverse, 5′-CCCCACTTGATTTTGGAGGGA-3′. The PCR products were analyzed through electrophoresis on a 1% agarose gel with an EcoDye™ Nucleic Acid Staining Solution (Biofact Co., Ltd., Daejeon, South Korea). Images were then captured using Wise Capture I-1000 software (Daihan Scientific, Seoul, South Korea).

### 4.5. Immunofluorescence Assay

RAW 264.7 cells were seeded onto a 4-well chamber slide (Lab-Tek#C7182) at a density of 3 × 10^3^ cells/mL. The cells were stimulated with LPS and then incubated with different concentrations of DA for the indicated duration. These were then washed with PBS and fixed in 3.7% formaldehyde for 10 min at room temperature. Afterwards, the cells were permeabilized with 0.1% Triton X-100 for 4 min and washed twice with PBS. Then, the cells were incubated with anti-CD11b, anti-iNOS, or anti-NFκB p65 antibodies (1:100 with 2% BSA) overnight at 4 °C, followed by incubation with goat anti-rabbit/anti-mouse IgG conjugated to either FITC or Alexa Flour 594 for 1 h at room temperature in the dark. Finally, the cells were washed with PBS and incubated with DAPI mounting medium for 5–10 min. The samples were then analyzed through fluorescence microscopy.

### 4.6. Enzyme-Linked Immunosorbent Assay (ELISA)

The levels of IL-1β and IL-6 exogenously secreted by the HL-60 and Raw 264.7 cells were determined through ELISA using a kit from Shire (Lexington, MA, USA). Ninety-six-well plates (MaxisorpImmuno-plate; Sigma-Aldrich, St. Louis, MO, USA) were coated with 10 μg/mL purified goat anti-IL-1β/anti-IL-6 antibodies. Cell culture supernatants from the control and treated groups were incubated in these plates for 1 h, and then a polyclonal rabbit anti-IL-1β/IL-6 antibody was added to the wells. HRP-conjugated goat anti-rabbit IgG was then added, and the cells were incubated for 30 min. 2,2′-Azino-bis(3-ethylbenzthiazoline-6-sulfonic acid) (ABTS, Sigma #A1888) was then added to the plate, and the absorbance at 405 nm was detected using a reference wavelength of 490 nm.

### 4.7. Western Blot

After cell lysis, the total protein content was isolated and quantified using the Bradford protein assay. The protein samples were denatured at 95 °C for 5 min. Next, the samples were analyzed through sodium dodecyl sulfate-polyacrylamide gel electrophoresis (SDS-PAGE) and Western blot using polyvinylidene fluoride microporous membranes. The membranes were incubated with primary antibodies (1:1000 with TBST) overnight at 4 °C, and then were incubated with HRP-conjugated secondary antibodies for 1 h. Protein bands were then detected using an ECL Western blotting detection reagent.

### 4.8. Statistical Analysis

All experiments were performed with triplicate samples, and all experiments were performed at least three times. Data are presented as the mean  ±  SD. Comparisons between two groups were analyzed using the Student’s *t*-test, while comparisons between more than two groups were analyzed using analysis of variance. Differences with *p* values ≤ 0.05 were considered statistically significant.
